# Spanish consensus on the management of concomitant antiseizure medications when using cenobamate in adults with drug‐resistant focal seizures

**DOI:** 10.1002/epi4.12936

**Published:** 2024-04-04

**Authors:** Mar Carreño, Antonio Gil‐Nagel, José M. Serratosa, Manuel Toledo, Juan Jesus Rodriguez‐Uranga, Vicente Villanueva

**Affiliations:** ^1^ Hospital Clinic Barcelona, Epilepsy Unit Barcelona Spain; ^2^ Department of Neurology, Epilepsy Program Ruber International Hospital Madrid Spain; ^3^ Department of Neurology Hospital Universitario Fundación Jiménez Díaz Madrid Spain; ^4^ Vall d'Hebron University Hospital, Epilepsy Unit Barcelona Spain; ^5^ Advanced Neurological Centre Seville Spain; ^6^ La Fe University and Polytechnic Hospital, Refractory Epilepsy Unit Valencia Spain

**Keywords:** antiseizure medication, cenobamate, dosage adjustment, epilepsy, titration

## Abstract

**Objective:**

Cenobamate is an antiseizure medication (ASM) associated with high rates of seizure freedom and acceptable tolerability in patients with focal seizures. To achieve the optimal cenobamate dose for maximal potential effectiveness while avoiding or minimizing drug‐related adverse events (AEs), the administration of cenobamate with other ASMs must be managed through concomitant ASM load reduction. A panel of Spanish epilepsy experts aimed to provide a Spanish consensus on how to adjust the dose of concomitant ASMs in patients with drug‐resistant epilepsy (DRE) in order to improve the effectiveness and tolerability of adjunctive cenobamate.

**Methods:**

A three‐stage modified Delphi consensus process was undertaken, including six Spanish epileptologists with extensive experience using cenobamate. Based on current literature and their own expert opinion, the expert panel reached a consensus on when and how to adjust the dosage of concomitant ASMs during cenobamate titration.

**Results:**

The expert panel agreed that tailored titration and close follow‐up are required to achieve the best efficacy and tolerability when initiating cenobamate in patients receiving concomitant ASMs. When concomitant clobazam, phenytoin, phenobarbital, and sodium channel blockers are taken at high dosages, or when the patient is receiving two or more sodium channel blockers, dosages should be proactively lowered during the cenobamate titration period. Other concomitant ASMs should be reduced only if the patient reports a moderate/severe AE at any stage of the titration period.

**Significance:**

Cenobamate is an effective ASM with a dose‐dependent effect. To maximize effectiveness while maintaining the best tolerability profile, co‐medication management is needed. The recommendations included herein provide practical guidance for proactive and reactive management of co‐medication in cenobamate‐treated patients with DRE and a high drug load.

**Plain Language Summary:**

Patients with epilepsy may continue to have seizures even after treatment with several different antiseizure medications (ASMs). Cenobamate is an ASM that can reduce seizures in these patients. In this study, six Spanish experts in epilepsy discussed the best way to use cenobamate in drug‐resistant epilepsy. They provide practical guidance on when and how the dose of other ASMs might be adjusted to reduce side effects and optimize the use of cenobamate.

**Key points**

- Cenobamate is associated with high seizure freedom rates and improved seizure control when used as adjunctive therapy.
- Drug–drug interactions between cenobamate and concomitant antiseizure medications (ASMs) may lead to adverse events.
- Dose adjustment of the concomitant ASMs is the primary way of maximizing cenobamate effectiveness while improving tolerability.
- Here we provide consensus recommendations from Spanish epilepsy experts for the management of concomitant ASMs in cenobamate recipients.

## 1 INTRODUCTION

The goal of epilepsy therapy is to achieve seizure freedom while minimizing clinically significant drug‐related adverse events (AEs).^1^ Treatment with monotherapy is preferred, and many patients can achieve seizure freedom with single‐drug therapy^1^, ^2^, ^3^; however, patients who do not achieve seizure freedom with monotherapy may require treatment with two or more antiseizure medications (ASMs). The risk/benefit ratio of polytherapy, in terms of efficacy, tolerability, and patient compliance, needs to be carefully evaluated. While some drug combinations can have clinically beneficial effects, unfavorable pharmacokinetic and pharmacodynamic interactions can lead to intolerable AEs in patients taking two or more concomitant ASMs.^4^

Despite the introduction of over a dozen new ASMs with different mechanisms of action into the market in the past 20 years, the long‐term outcomes in adult patients diagnosed with epilepsy have not substantially improved,^2^, ^5^ and over a third of patients have persistent drug‐resistant epilepsy (DRE). Thus, there is still a need for novel effective ASMs with acceptable tolerability to achieve seizure freedom. Cenobamate, an orally administered tetrazole alkyl carbamate derivative, is approved in the United States (US) for the treatment of focal‐onset seizures in adult patients as adjunctive therapy and as monotherapy,^6^ and in Europe for the adjunctive treatment of focal‐onset seizures with or without secondary generalization (now known as focal to bilateral tonic–clonic seizures) in adult patients with epilepsy who have not been adequately controlled despite a history of treatment with at least two ASMs.^7^ Cenobamate has a dual mechanism of action, enhancing gamma‐aminobutyric acid (GABA) activity through positive allosteric modulation of the GABA‐A receptor, binding at a different site to the benzodiazepine binding site,^8^ and attenuating the persistent sodium current of sodium channels.^9^ As adjunctive therapy in clinical trials, cenobamate has been associated with high seizure freedom rates and improved seizure control, high retention rates, and acceptable tolerability.^10^, ^11^, ^12^, ^13^, ^14^

In vitro studies have shown that cenobamate inhibits cytochrome P450 (CYP)2C19 and induces CYP3A4 and CYP2B6^7^; as a result, cenobamate may increase exposure to agents primarily metabolized by CYP2C19 and reduce exposure to agents metabolized by CYP3A4 and CYP2B6.^7^ Drug–drug interactions have been reported between cenobamate and phenytoin, phenobarbital, clobazam, and lamotrigine, such that the dose of cenobamate or the concomitant ASM may need to be adjusted to ensure correct exposure and unchanged efficacy.^7^ Drug–drug interactions between cenobamate and other ASMs may also lead to AEs; for example, significant somnolence has been reported with concomitant administration of cenobamate and clobazam,^15^ and an increase in other central nervous system AEs such as dizziness has been reported when cenobamate is combined with other ASMs that inhibit sodium channels.^16^, ^17^, ^18^

Evidence from clinical trials and real‐world studies suggests that most adverse impacts on patients receiving cenobamate in combination with other ASMs can be effectively managed by decreasing the dose of, or completely discontinuing, the concomitant ASMs.^19^, ^20^, ^21^, ^22^ Tapering of concomitant ASMs is an acknowledged method of reducing AEs in patients taking multiple ASMs including cenobamate.^17^ Reducing the dose or discontinuing concomitant ASMs has been shown to resolve AEs spontaneously in some patients,^20^ while not doing so is thought to exacerbate AEs.^18^ Recent consensus articles by epilepsy experts in the US and Italy with experience using cenobamate recommend proactively (prior to report of an AE) and reactively (in response to report of an AE) lowering the dose of specific concomitant ASMs during cenobamate titration to prevent or mitigate AEs.^23^, ^24^

This study aimed to provide a consensus on how to adjust the dose of concomitant ASMs in drug‐resistant patients who typically have a high drug load to improve the effectiveness and tolerability of adjunctive cenobamate.

## 2 METHODS

A panel consisting of six specialist physicians, who were selected based on their expertise in epilepsy and extensive experience with cenobamate during its clinical development and/or the early access program, as well as in managing patients with other ASMs, was convened by Angelini Pharma. Angelini Pharma did not have any involvement in the direction of the panel's recommendations or in the approval of the submitted manuscript.

**FIGURE 1:**
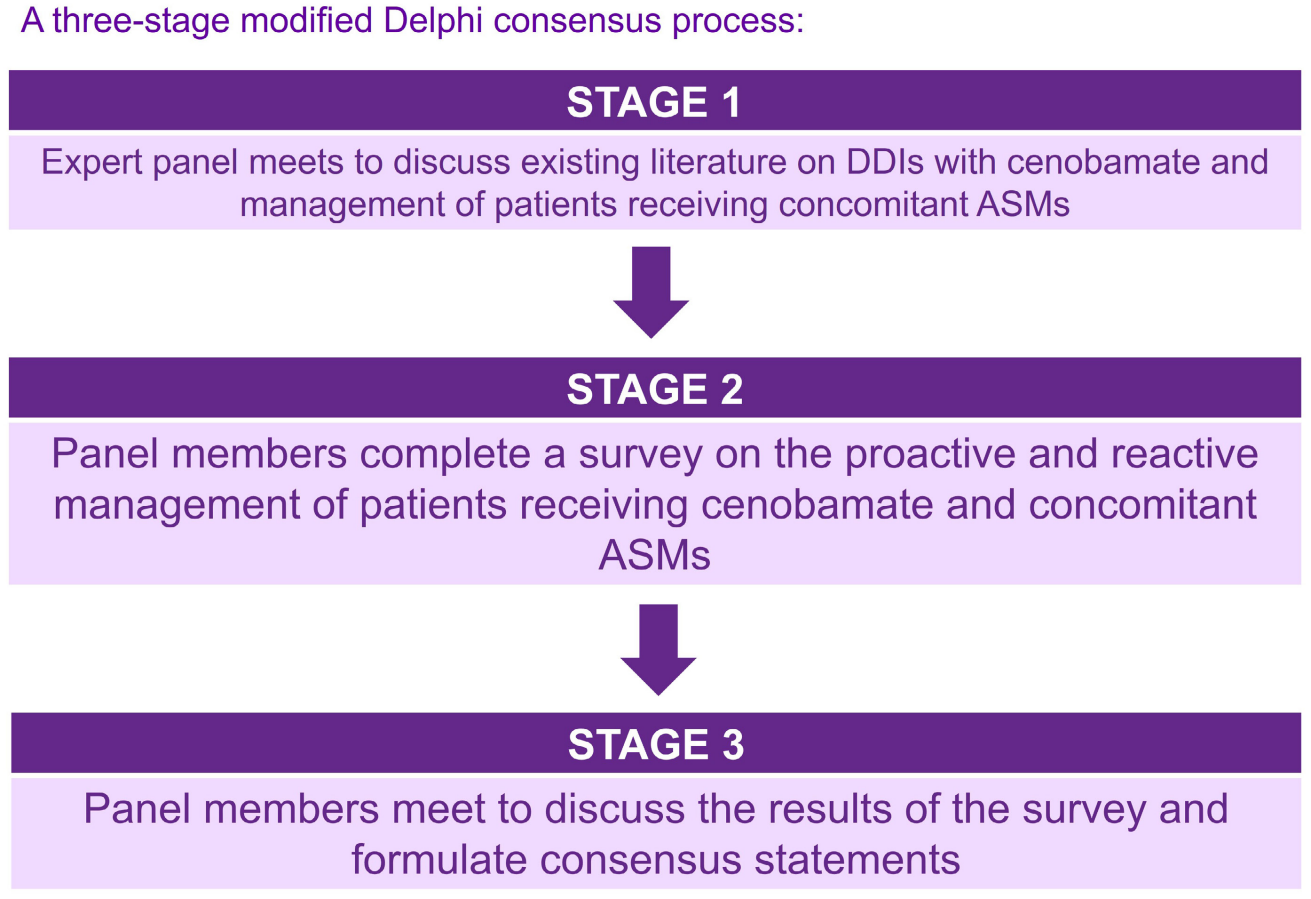
The modified Delphi method followed by the expert panel. ASMs, antiseizure medications; DDIs, drug–drug interactions.

A three‐stage modified Delphi process was used to develop recommendations on the use of cenobamate as adjunctive therapy in adults with drug‐resistant focal‐onset seizures (Figure 1). Stage 1 consisted of a face‐to‐face meeting of the expert panel to discuss available published data on the pharmacokinetic and pharmacodynamic profile of cenobamate and existing recommendations for the management of concomitant ASMs in patients receiving cenobamate. In stage 2, panelists completed a survey based on the known drug interaction profile of cenobamate, including questions regarding the proactive and/or reactive management of patients receiving cenobamate and concomitant sodium channel blockers (SCBs; carbamazepine, oxcarbazepine, eslicarbazepine acetate, lacosamide, lamotrigine; proactive management: 10 questions, reactive management: 14 questions), GABAergic drugs (benzodiazepines, gabapentin, pregabalin, clobazam, tiagabine; proactive management: 12 questions, reactive management: 11 questions), drugs with multiple mechanisms of action (topiramate, valproate; proactive management: 5 questions, reactive management: 0 questions) and other (perampanel, levetiracetam, brivaracetam, zonisamide, phenytoin and phenobarbital; proactive management: 13 questions, reactive management: 0 questions). The survey questions are summarized in the Table S1. Panelists were asked to answer the questions based on previously published studies and their experience in clinical practice. Stage 3 was a hybrid meeting where all the survey answers were shared with the panelists and discussed in detail until a statement that all panelists agreed with was formulated.

As the project was limited to a survey and discussion of factors affecting therapeutic practice, Ethics Committee approval was not required.

## 3 RESULTS

### 3.1 General considerations

The expert panel recommended that cenobamate should be initiated and titrated as per the approved label, starting at 12.5 mg/day and increasing the dose every 2 weeks to 25, 50, 100, 150, and 200 mg/day. The initial target dose should be 200 mg/day and, based on the clinical response, the dose may be increased to a maximum of 400 mg/day.

Patients with highly drug‐resistant focal epilepsy might benefit from a higher dose of cenobamate (>200 mg). Slower titration does not appear to improve tolerability, as seen in clinical trials and real‐world evidence,^21^, ^25^ while co‐medication management appears to be a key aspect of improving tolerability^20^, ^21^, ^22^; nevertheless, individualization of therapy is recommended.

Regarding adjusting the dose of concomitant ASMs, the panel agreed that proactive dose reduction of concomitant ASMs is not required in every patient starting cenobamate. When making a proactive adjustment to the doses of concomitant ASMs, factors that should be considered include the types of concomitant ASMs, how well the concomitant ASMs are being tolerated, any comorbidities present, and disease severity and seizure type. Generally, patients with DRE and a high drug load (i.e., patients taking multiple concomitant ASMs or ASMs at high dosages, resulting in a high total drug load) might benefit from drug load reductions. In contrast, patients with a lower drug load (e.g., patients who use cenobamate earlier in their treatment pathway) may need less or no co‐medication management. If dose reduction is necessary, the panel advises reducing concomitant ASMs gradually as the dose of cenobamate is increased to minimize any adverse impacts of medication withdrawal.

### 3.2 Patients receiving concomitant sodium channel blockers

**FIGURE 2:**
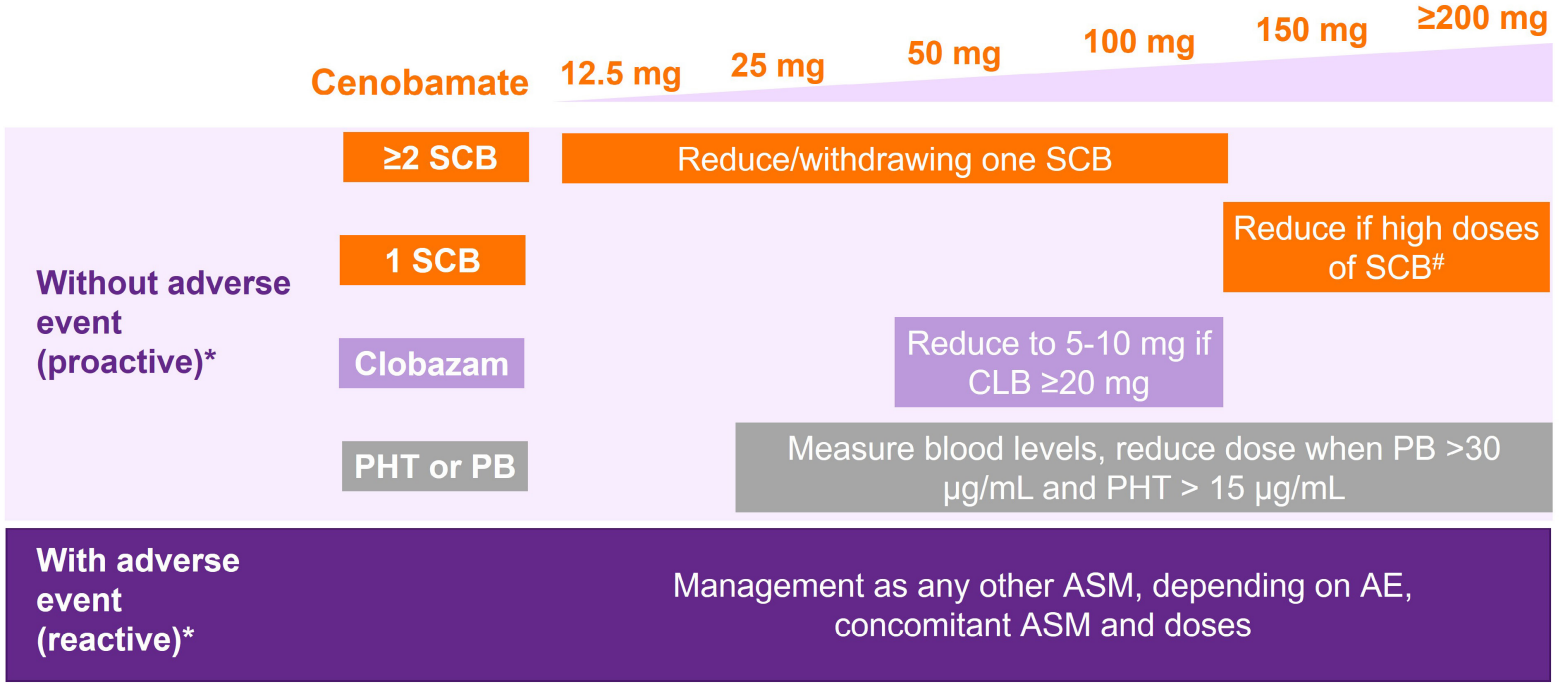
Summary of expert recommendations for adjusting the dose of concomitant ASMs when initiating cenobamate. #Carbamazepine ≥800 mg/day, oxcarbazepine >900 mg/day, eslicarbazepine >1200 mg/day, lacosamide >300 mg/day, and lamotrigine >300 mg/day; *On a case‐by‐case basis, considering type of ASM, doses, blood levels, tolerability to ASMs, patients' comorbidities, disease severity, type and frequency of seizures. AEs, adverse events; ASMs, antiseizure medications; CLB, clobazam; PB, phenobarbital; PHT, phenytoin; SCB(s), sodium channel blocker(s).

In patients receiving two or more SCBs when initiating cenobamate, the expert panel suggests proactively reducing the dose/withdrawal of at least one of the SCBs once the dosage of cenobamate reaches 12.5–100 mg/day and before AEs appear, taking into consideration disease severity and the tolerability and dose of the concomitant SCBs (Figure 2).

In patients receiving high‐dose SCBs (defined as carbamazepine ≥800 mg/day, oxcarbazepine >900 mg/day, eslicarbazepine >1200 mg/day, lacosamide >300 mg/day, and lamotrigine >300 mg/day) when initiating cenobamate, the panel recommends proactively reducing the SCB dose once the dose of cenobamate reaches ≥150 mg/day to avoid or minimize side effects. High‐dose SCBs should be decreased slowly to prevent any increase in seizure risk.

AEs should be managed reactively in patients who experience moderate daytime somnolence or dizziness for ≥7 days by reducing the dose of concomitant SCBs. In the event of severe somnolence or dizziness, the SCB dose should be reduced sooner. SCB dose should also be reduced if patients report persistent diplopia for ≥3 days. If the AEs are mild but persistent, and impact activities of daily living, the panel recommends making a slight reduction to the dose of the concomitant SCB or changing the time of administration (for example, suggesting bedtime dosing if the patient is experiencing daytime somnolence).

### 3.3 Patients receiving concomitant GABAergics

In the case of GABAergic drugs, special attention should be given to benzodiazepines and benzodiazepine‐derived drugs, and to CLB in particular due to the pharmacokinetic and pharmacodynamic interactions reported with cenobamate.^15^, ^26^, ^27^

In patients taking clobazam ≥20 mg/day, proactive dose reduction should be considered when the administered dose of cenobamate reaches 50–100 mg/day to avoid the risk of drowsiness (Figure 2). However, they recommend not completely withdrawing clobazam, as in some patients, a low dose of clobazam (5–10 mg/day) in combination with cenobamate could exert a beneficial effect on seizure control.^15^, ^22^, ^28^

AEs in patients receiving clobazam should be managed reactively (Figure 2). If patients receiving concomitant clobazam and one SCB report drowsiness, the dose of clobazam should be decreased; if these patients report dizziness, the dose of SCB should be decreased or further decreased. Regarding other concomitant GABAergic drugs, doses should be reduced in patients reporting moderate daytime somnolence or fatigue lasting ≥7 days. If the reported AEs are mild but persistent, and impact activities of daily living, the panel advises considering a slight reduction to the dose of GABAergic drug or changing the time of administration.

### 3.4 Patients receiving concomitant phenytoin and/or phenobarbital

The expert panel suggests proactively reducing the dose of phenytoin if the blood level is >15 μg/mL when cenobamate has reached a dose of 25 mg/day (Figure 2). They also recommend always measuring the blood levels of phenytoin, or at least if phenytoin‐related AEs (dizziness, somnolence, ataxia, diplopia) appear during cenobamate titration. Once the dosage is stabilized, no routine blood level measurements are needed.

Similarly, in patients taking concomitant phenobarbital, the panel suggests measuring blood levels and proactively reducing the dose of phenobarbital if levels are >30 μg/mL with doses of cenobamate ≥25 mg/day (Figure 2). If phenobarbital‐related AEs (somnolence, fatigue) appear during cenobamate titration, blood levels of phenobarbital should be measured again. Once the dosage is stabilized, no routine blood level measurements are needed.

Due to the narrow therapeutic index of both phenytoin and phenobarbital, special care should be taken with patients taking these agents with other SCB or GABAergic ASMs due to pharmacokinetic and pharmacodynamic interactions with cenobamate.

### 3.5 Patients receiving other concomitant ASMs

In patients who are receiving concomitant perampanel, levetiracetam, brivaracetam, or zonisamide, the panel recommends decreasing the dose of these drugs reactively, in response to the type of AE reported (Figure 2). In patients who are receiving an SCB plus one of these other ASMs, dose adjustments of the SCB or the other ASM should be made according to the AE reported.

### 3.6 Patients receiving two or more concomitant ASMs with different mechanisms of action

In patients taking two or more concomitant ASMs with different mechanisms of action (topiramate or valproate), neurologists should consider reactive dose reductions depending on the type of AEs reported, concomitant ASM doses, and the specific recommendations provided above and in Figure 2. In addition to prior recommendations in patients reporting somnolence and fatigue, the experts suggest reducing the doses of synaptic vesicle 2A membrane protein (SV2A) modulators (levetiracetam or brivaracetam) first.

## 4 DISCUSSION

Cenobamate is a dual GABAergic/SCB ASM associated with high seizure freedom rates and improved seizure control.^10^, ^11^, ^12^, ^13^, ^14^ In one real‐world study of cenobamate in highly drug‐resistant patients (≥4 previous ASMs), all treatment discontinuations occurred in patients who were receiving ≥3 concomitant ASMs^22^; therefore, in order to maximize the effectiveness and tolerability of cenobamate, patients with DRE and a high drug load might benefit from reducing these concomitant ASMs. This may also apply to patients who are receiving first or second‐generation ASMs that are generally used as second‐line options. Existing literature provides some data on how multidrug ASM regimens, including cenobamate, can be managed; for example, in patients with highly active and drug‐resistant focal epilepsy, the most commonly reported AE with cenobamate, daytime somnolence, can be managed by reducing the dose of concomitant clobazam, eslicarbazepine, and perampanel.^20^, ^22^ In a cohort of patients with drug‐resistant focal epilepsy in routine clinical practice, the use of cenobamate allowed for a significant decrease in concomitant ASM use, with treatment‐emergent AEs resolving or improving in 85% of patients.^21^ However, when the majority of evidence potentially impacting the management of patients is available across multiple publications, it is of interest to provide some easy‐to‐use consensus guidelines for neurologists managing patients with epilepsy who are receiving multidrug regimens.

The current publication summarizes recommendations provided by six epilepsy specialists with extensive experience in the use of cenobamate in Spain. The panelists agreed that when adding cenobamate as adjunctive therapy, especially in highly DRE patients and those treated with several concomitant ASMs, the doses of clobazam, phenytoin, phenobarbital and high‐dose SCBs should be proactively lowered during the titration period to prevent potential AEs caused by pharmacokinetic or pharmacodynamic interactions among the ASMs. It is important to emphasize that tolerability of different ASMs can vary substantially between patients and is dependent on individual patient characteristics. Therefore, a standardized methodology for dose adjustments cannot be implemented for all patients. Dose adjustments should therefore be made on a *case‐by‐case basis*, bearing in mind the concomitant ASM doses, seizure type and frequency, tolerability to ASMs, and comorbidities. Other concomitant ASMs should be reactively reduced if the patient reports a moderate/severe AE at any stage of the cenobamate titration period. In the choice of which ASM to lower, the type of AE and concomitant ASM doses should be considered. The experts would preferentially reduce the dose of an SCB in a patient reporting dizziness or diplopia, whereas if a patient reports somnolence or fatigue, they would tend to reduce the dose of GABAergic ASMs or SV2A modulators.

These recommendations are based on the experience of the experts with the Spanish population and are not as prescriptive as those made by epileptologists in the US,^23^ where higher ASM doses are usually used. Different approaches to clinical practice management for cenobamate, especially in terms of dosage of concomitant medications, between the US and Europe may reflect both different population characteristics and different indications for cenobamate. Generally, the US experts recommended proactive adjustment of concomitant high‐dose ASMs, similar to the Spanish recommendations, but what is considered high dose differs^23^; for example, recommendations by the Spanish experts consider high dose to be ≥800 mg/day carbamazepine and >300 mg/day lamotrigine, versus >1200 mg/day and 500 mg/day, respectively, in the US recommendations. In addition, the dose of cenobamate at which doses of concomitant ASMs can begin to be adjusted is also higher in the US recommendations^23^ (150–200 mg/day compared with 25–50 mg/day for most of the Spanish recommendations). Finally, while the American consensus only gives proactive recommendations with high doses of lacosamide, our recommendations are for those receiving two SCBs or high‐dose SCBs.

Italian consensus recommendations are also available.^24^ Compared with the Italian consensus, the Spanish consensus provides more detailed information on concomitant medication, including management and dosage. Recommendations to improve tolerability of cenobamate in patients with epilepsy by the Italian experts included a slower titration, dosing in the evening, and lowering the dose of concomitant ASMs, particularly in patients receiving high‐dose concomitant ASMs (and especially SCBs) or benzodiazepines. Some of these recommendations are similar to those outlined by the Spanish experts. For example, the cenobamate dose at which Italian experts started to lower concomitant ASMs was 100 mg/day regardless of the occurrence of AEs, an approach more similar to the Spanish experts than the US experts.^23^, ^24^ On the other hand, while the Italian experts also acknowledged that seizure freedom could sometimes be achieved with doses of cenobamate as low as 50–100 mg/day,^24^ we recommend co‐medication be managed in order to achieve the maximal potential effectiveness. Last, according to our experience, slower titration does not appear to improve tolerability, and this is supported by data from clinical trials and real‐world evidence^21^, ^25^; however, individualizing therapy is recommended. As in other therapeutic scenarios with ASMs, when achieving seizure freedom after the addition of cenobamate, the risks and benefits of transitioning to monotherapy could be considered. Further real‐world data and open‐label studies are needed to determine whether seizure freedom can be maintained with cenobamate monotherapy.^29^ Promising results in patients taking cenobamate and low‐dose clobazam^15^, ^22^ suggest that seizure freedom can be maintained with a low number of ASMs.

As greater efficacy in reducing seizure frequency has been reported with higher doses of cenobamate (200–400 mg/day),^30^ effectively managing cenobamate‐related AEs will help to achieve further improvements in seizure control in patients who require high cenobamate doses. Future studies on the interactions between cenobamate and concomitant medications, not just ASMs, will contribute to maximizing the therapeutic benefits of cenobamate while minimizing the risk of AEs.

## 5 CONCLUSIONS

Cenobamate has been shown to be very effective in reducing focal seizures in patients. Based on the experience of six Spanish epilepsy experts, this article provides practical guidance for the use of cenobamate in patients with epilepsy treated with a multidrug ASM regimen. Co‐medication management is generally required to achieve the best effectiveness and tolerability during the titration and maintenance period, especially in patients with DRE and a high drug load. While the doses of clobazam, phenytoin, phenobarbital, and SCBs might have to be reduced proactively during cenobamate titration, especially if the patient takes high doses of SCBs or more than one SCB, other concomitant ASMs should be reduced reactively according to the type of AEs.

## AUTHOR CONTRIBUTIONS

All authors contributed equally. All authors discussed the results, revised the first draft, and contributed to the final manuscript. All authors have read and approved the submitted version of the manuscript and accept the responsibility for its content.

## FUNDING INFORMATION

This research was funded by Angelini Pharma.

## CONFLICT OF INTEREST STATEMENT

MC has received honoraria for advisory, educational activities, and/or research funds from Angelini Pharma, Bial, Eisai, GW Pharmaceuticals, and UCB Pharma; AGN has received honoraria for advisory, educational activities, and/or research funds from EISAI, UCB Pharma, Bial, Esteve, Jazz Pharma, Sanofi, Novartis, Roche, and Angelini Pharma; JMS has received honoraria for advisory, educational activities, and/or research funds from Angelini Pharma, BIAL, Eisai Inc, Esteve, Ferrer, Jazz Pharmaceuticals, GW Pharmaceuticals, Sanofi, UCB Pharma, and Zogenix. MT has received honoraria for advisory, educational activities, and/or research funds from Angelini Pharma, Bial, Eisai Inc, GSK, GW Pharmaceuticals, and UCB Pharma. JRA has received honoraria for advisory, educational activities, and/or research funds from Eisai, GW Pharma, Jazz Pharmaceutical Iberia, UCB Pharma, Bial, Novartis, Livanova, Angelini Pharma, GlaxoSmithKline, Pfizer, and Esteve. VV has received honoraria for advisory, educational activities, and/or research funds from Angelini Pharma, Bial, Biocodex, Eisai Inc, Jazz Pharmaceuticals, Novartis, Takeda, UCB Pharma, and Xenon. We confirm that we have read the Journal's position on issues involved in ethical publication and affirm that this report is consistent with those guidelines.

## ETHICS STATEMENT

This project was limited to a survey and discussion of factors affecting therapeutic practice, so Ethics Committee approval was not required.

## PATIENT CONSENT STATEMENT

Not applicable.

## Supporting information


Table S1

## Data Availability

No data sets were generated during the preparation of this manuscript. All information used was either in the public domain or was part of the authors' clinical experience.
