# Supplementary material for: Spanish consensus on the management of concomitant antiseizure medications when using cenobamate in adults with drug‐resistant focal seizures
Source: Epilepsia Open. 2024 Apr 4;9(3):1051–8. doi: 10.1002/epi4.12936 (PMC11145622; doi:10.1002/epi4.12936)
Supplement: Supplementary file 1 — Table S1 [file EPI4-9-1051-s001.docx]

# Carreño et al. Supporting Information

## Table S1.

Survey completed by the expert panellists

| **Survey questions** | **Yes/No (required)** | **DRUG /DOSE**  **(only when answer is YES)** | **Comments** |
| --- | --- | --- | --- |
| **Sodium channel blockers** |  |  |  |
| I proactively reduce the dose of SCBs in: |  |  |  |
| Any patient |  |  |  |
| Any patient with ≥2 concomitant ASMs |  |  |  |
| Any patient with ≥2 concomitant SCB |  |  |  |
| Any patient with concomitant SCB and ….. |  |  |  |
| Any patient taking carbamazepine over the dose of |  |  |  |
| Any patient taking oxcarbazepine over the dose of |  |  |  |
| Any patient taking eslicarbazepine over the dose of |  |  |  |
| Any patient taking lacosamide over the dose of |  |  |  |
| Any patient taking lamotrigine over the dose of |  |  |  |
| Others (please add in comments) |  |  |  |
| I reactively reduce the dose of SCB, when side effects appear: |  |  |  |
| Any side effect |  |  |  |
| Any duration of side effect |  |  |  |
| Any severity |  |  |  |
| Any time a patient complains about side effects |  |  |  |
| Always when somnolence appears |  |  |  |
| Always when somnolence appears for more than X days |  |  |  |
| Always when somnolence appears to be (mild/moderate/severe) |  |  |  |
| Always when dizziness appears |  |  |  |
| Always when dizziness appears for more than X days |  |  |  |
| Always when dizziness appears to be (mild/moderate/severe) |  |  |  |
| Always when diplopia appears |  |  |  |
| Always when diplopia appears for more than X days |  |  |  |
| Always when diplopia appears to be (mild/moderate/severe) |  |  |  |
| Others (please add in comments) |  |  |  |
| **GABAergic drugs** |  |  |  |
| I proactively reduce the dose of GABAergic in: |  |  |  |
| Any patient |  |  |  |
| Any patient with ≥2 concomitant ASMs |  |  |  |
| Any patient with ≥2 concomitant GABAergic drugs |  |  |  |
| Any patient with ≥2 concomitant GABAergic drugs if one is a benzodiazepine |  |  |  |
| Any patient taking |  |  |  |
| Any patient taking gabapentin over the dose of |  |  |  |
| Any patient taking pregabalin over the dose of |  |  |  |
| Any patient taking tiagabine over the dose of |  |  |  |
| Any patient taking benzodiazepines |  |  |  |
| Any patient taking clobazam |  |  |  |
| Any patient taking clobazam over the dose of |  |  |  |
| Others (please add in comments) |  |  |  |
| I reactively reduce the dose of GABAergic, when side effects appear |  |  |  |
| Any side effect |  |  |  |
| Any duration of side effect |  |  |  |
| Any severity |  |  |  |
| Any time a patient complains about side effects |  |  |  |
| Always when somnolence appears |  |  |  |
| Always when somnolence appears for more than X days |  |  |  |
| Always when somnolence appears to be (mild/moderate/severe) |  |  |  |
| Always when dizziness appears |  |  |  |
| Always when dizziness appears for more than X days |  |  |  |
| Always when dizziness appears to be (mild/moderate/severe) |  |  |  |
| Others (please add in comments) |  |  |  |
| **Drugs with multiple MoAs (SCB + GABA)** |  |  |  |
| I proactively reduce the dose of drugs with multiple MoAs: |  |  |  |
| Any patient taking topiramate |  |  |  |
| Any patient taking topiramate over the dose of … |  |  |  |
| Any patient taking valproate |  |  |  |
| Any patient taking valproate over the dose of … |  |  |  |
| Others (please add in comments) |  |  |  |
| **Other drugs** |  |  |  |
| I proactively reduce the dose of: |  |  |  |
| Any patient taking perampanel |  |  |  |
| Any patient taking perampanel over the dose of … |  |  |  |
| Any patient taking levetiracetam |  |  |  |
| Any patient taking levetiracetam over the dose of … |  |  |  |
| Any patient taking brivaracetam |  |  |  |
| Any patient taking brivaracetam over the dose of … |  |  |  |
| Any patient taking zonisamide |  |  |  |
| Any patient taking zonisamide over the dose of … |  |  |  |
| Any patient taking phenytoin |  |  |  |
| Any patient taking phenytoin over the dose of … |  |  |  |
| Any patient taking phenobarbital |  |  |  |
| Any patient taking phenobarbital over the dose of … |  |  |  |
| Others (please add in comments) |  |  |  |
| **Additional questions** |  |  |  |
| When I proactively recommend to reduce concomitant ASMs, I start at the following dose of cenobamate |  |  |  |
| If previous answer = "depends" please explain in comments |  |  |  |
| **Additional considerations** (for free text comments) |  |  |  |

ASM, antiseizure medication; GABA, gamma-aminobutyric acid; MoAs, mechanisms of action; SCB, sodium channel blocker.
